# Atomistic Observation of Defect Generation and Microstructural Evolution in Polycrystalline FeCrAl Alloys Under Different Irradiation Conditions

**DOI:** 10.3390/nano15130988

**Published:** 2025-06-26

**Authors:** Huan Yao, Changwei Wu, Tianzhou Ye, Pengfei Wang, Junmei Wu, Yingwei Wu, Ping Chen

**Affiliations:** 1 Comac Aviation College, Ordos Institute of Technology, No. 1 East Ordos Street, Kambashi County, Ordos 017000, China; yaohuan@oit.edu.cn; 2 School of Aerospace, Xi’an Jiaotong University, No. 28, Xianning West Road, Beilin County, Xi’an 710049, China; changweiwu@stu.xjtu.edu.cn; 3 School of Nuclear Science and Technology, Xi’an Jiaotong University, No. 28, Xianning West Road, Beilin County, Xi’an 710049, China; mytz123@stu.xjtu.edu.cn (T.Y.); wyw810@mail.xjtu.edu.cn (Y.W.); 4 Nuclear Power Institute of China, No. 328 Huayang Changshun Avenue Section 1, Shuangliu County, Chengdu 610213, China; wpf03082108@163.com (P.W.); chenping_npic@163.com (P.C.)

**Keywords:** neutron irradiation, structural evolution, damage dose, molecular dynamics

## Abstract

FeCrAl alloys have garnered considerable attention as candidate cladding materials for light water reactors due to their promising mechanical stability and irradiation resistance. However, the response characteristics of these alloys to irradiation and the associated mechanisms remain poorly understood. This study provides atomistic insights into irradiation-induced defect formation and microstructural evolution in polycrystalline FeCrAl. Using the LAMMPS molecular dynamics code, displacement cascades were simulated under irradiation doses ranging from 0.05 dpa to 0.5 dpa while evaluating the dependencies on temperature and grain size. The interaction between pre-existing defects and irradiation-induced microstructures (point defects, dislocations, clusters, etc.) was visualized and analyzed visually and quantitatively. The results indicate that the irradiation dose increases the number of surviving Frenkel pairs, whereas elevated temperatures reduce their stability. The cluster fraction of interstitials increases with both irradiation dose and temperature, while that of vacancies decreases at higher temperatures due to their lower stability. In the initial phase of the displacement cascade, the density and distribution of dislocations evolve continuously until the annealing stage. The dislocation density at the end of the annealing phase decreases with increasing dose and temperature. The thickness of grain boundaries increases with the irradiation dose, and the regions adjacent to grain boundaries transform into an amorphous state at higher dose levels. As both the irradiation dose and temperature increase, the amorphization process accelerates, and smaller grain size leads to a greater degree of amorphization.

## 1. Introduction

Nuclear energy plays a critical role in addressing global energy challenges due to its cleanliness, sustainability, cost-effectiveness, and high efficiency [1]. Following the Fukushima nuclear accident in 2011, the international nuclear industry recognized the urgent need for the research and development of accident-tolerant fuel (ATF) [2]. ATF aims to provide extended response time to mitigate potential future accidents. The cladding of fuel rods serves as the primary barrier to prevent the release of fission products in high-temperature and high-radiation environments [3]. Zircaloy is commonly employed as cladding material for light water reactors (LWRs) in nuclear power plants [4]; however, it reacts with high-temperature steam, producing hydrogen and potentially causing hydrogen explosions [5]. FeCrAl, an advanced iron-based superalloy, has been proposed as a candidate cladding material for ATF due to its superior mechanical strength and irradiation resistance [6,7,8,9]. FeCrAl alloys have demonstrated comparable or enhanced performance relative to zircaloy under both normal operating and accident conditions [10,11].

Under neutron irradiation, FeCrAl alloys are subjected to bombardment by energetic neutrons, and neutrons will slow down and transfer part of their initial energy to lattice atoms, generating primary knock-on atoms (PKAs) [12]. These PKAs initiate displacement cascades and sub-cascades through collisions with lattice atoms, resulting in the formation of numerous vacancy–interstitial Frenkel pairs (FPs) [13]. Although displacement cascades occur on the picosecond timescale, the resulting defects lead to significant material degradation, including swelling, hardening, embrittlement, and irradiation creep, which limit the service life of FeCrAl cladding in ATF applications [14]. Therefore, investigating the characteristics of radiation-induced damage in FeCrAl alloys is essential, with particular emphasis on the formation and evolution of defect structures under neutron irradiation.

Experimental studies on the irradiation behavior of FeCrAl alloys remain relatively limited due to high costs, long cycle durations, and intense radioactivity. In FeCr alloys [15,16,17], an increase in Cr content from 10 to 12 wt% significantly reduces the dislocation loop size and increases their number; however, further increases beyond 12 wt% exert minimal influence. In this regard, FeCrAl alloys with high Cr content exhibit exceptional radiation tolerance, even under extreme high-temperature and high-irradiation conditions. For the FeCrAl alloy system, the primary radiation damage is attributed to the formation of dislocation loops, dislocation lines, defect clusters, and Cr-rich precipitates. Studies have shown that pre-existing dislocation loops in FeCrAl can be eliminated [10]; however, new dislocation loops form at the grain boundary (GB) under irradiation, leading to increased dislocation density post-irradiation [18]. Yuchi et al. [19] observed that the radiation-induced hardening of FeCrAl is primarily attributed to the increased slip resistance caused by radiation-generated dislocation loops. Cr precipitates are known to contribute to hardening and embrittlement effects [20,21]. Kobayashi et al. [22] reported that elevated Al content suppresses Cr precipitate formation, thereby mitigating irradiation-induced embrittlement.

Molecular dynamics (MD) simulations have emerged as a powerful computational approach to reduce the time and cost associated with experimental irradiation studies. Using the MD method, researchers can efficiently investigate the microstructural evolution of FeCrAl alloys under various irradiation conditions. In recent years, several studies have employed simulations to analyze displacement cascades in α-Fe. Sahi et al. [23] and Zhang et al. [24] examined displacement cascades in single-crystal (SC) Fe and nanowires, respectively, focusing on the effects of PKA directions and energies on point defect behavior. Carlos et al. [25] conducted similar simulations on α-Fe and further explored the influence of displacement cascades on GB structure. These studies found that irradiation-induced point defects preferentially accumulate at GBs and induce GB motion through sliding or migration mechanisms. Prior research has demonstrated that Cr addition significantly enhances the radiation resistance of FeCr alloys compared to pure α-Fe [26,27]. Malerba and Terentyev et al. [28,29] investigated irradiation damage in Fe–Cr alloys, revealing that Cr in interstitial clusters impedes the mobility of dislocation loops. Furthermore, the addition of Al contributes to the improved mechanical properties of FeCrAl alloys [30]. Recent MD simulations have elucidated the fundamental mechanisms of FP generation and evolution during displacement cascades in SC FeCrAl [30]. Compared to pure α-Fe, the Cr and Al alloying elements in FeCrAl increase the number of surviving FPs while reducing the number of defect clusters formed during the annealing phase. Zheng [31] observed that, under conditions of high temperature and high-energy irradiation, large-sized interstitial clusters and a small number of vacancy clusters are more likely to form in SC FeCrAl alloys. The work mentioned above underscores the value of MD simulation in providing new and complementary insights into the behavior and mechanisms of irradiation for FeCrAl alloys, which are not readily available from experiments. Moreover, it is important to note that GBs may significantly influence the number and distribution of defects during displacement cascades. However, atomic-scale simulations of displacement cascades in polycrystalline (PC) FeCrAl remain scarce, despite a few experimental investigations into the irradiation behavior of coarse-grained FeCrAl alloys.

In the present study, a nanostructured model of PC FeCrAl was constructed, and displacement cascade simulations were conducted using LAMMPS under various irradiation doses ranging from 0.05 dpa to 0.5 dpa. The number, distribution, and morphology of FP defects, along with the effects of irradiation dose, temperature, and grain size (GS), were preliminarily analyzed. The fractions of clustered interstitial and vacancy defects were further evaluated quantitatively. Additionally, the temporal evolution of dislocation defects, including their density and distribution, as well as the formation and evolution of GB and amorphous structures, was investigated. Based on the analyses, the primary mechanisms influencing the irradiation behavior of PC FeCrAl alloys are discussed in detail.

## 2. Model and Methodology

### 2.1. Atomic-Scale Model of PC FeCrAl Alloy

**Figure 1:**
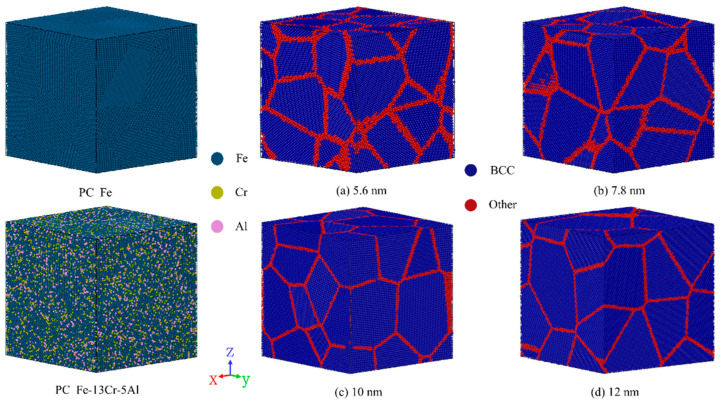
Polycrystalline (PC) Fe-13Cr-5Al models characterized by the local structural environment of atoms using the common neighbor analysis (CNA) method.

It is widely recognized that FeCrAl alloys exist as an Fe-based solid solution with a body-centered cubic (BCC) structure [32]. Cr and Al are considered to be uniformly and randomly distributed at the lattice positions of Fe atoms [33,34]. Previous studies showed that an alloy composition of 13 wt% Cr and 5 wt% Al provides favorable manufacturing, processing, mechanical, and thermophysical properties for FeCrAl fuel cladding applications [21]. In this study, the atomic-scale models of PC Fe with random positions and orientations were constructed using the software Atomsk Beta 0.13.1 [35], an open-source tool for generating FCC/BCC lattices, introducing defects (e.g., vacancies, interstitials, and dislocations) and building polycrystals. Subsequently, Fe lattice atoms were randomly substituted with Cr and Al atoms using Python 3.13 scripts to construct PC Fe-13Cr-5Al models. Figure 1 displays PC Fe-13Cr-5Al simulation models with GSs of 5.6, 7.8, 10, and 12 nm, respectively. In the figure, GBs and intragranular regions were distinguished by crystal structure using the common neighbor analysis (CNA) method [36]. To optimize computational resources, the number of grains decreases as GS increases. Table 1 summarizes the parameters of the PC Fe-13Cr-5Al models.

### 2.2. Simulation Method and Conditions

In physical systems, irradiation-induced collision cascades result in localized energy non-conservation due to the rapid dissipation of kinetic energy. The cascade center is extensively heated as the kinetic energy of involved atoms increases significantly, and this energy is transferred to surrounding atoms through thermal conduction. In MD simulations, cascade modeling typically employs small simulation cells with edge lengths of 10–100 nm and atom counts ranging from 10^5^ to 10^7^. In comparison, FeCrAl cladding with a diameter of 9.0 mm and a length of 1000 mm may contain up to 10^25^ atoms.

Although coarse-grained models offer superior computational efficiency and permit access to extended temporal and spatial scales (up to or close to 10^25^ atoms), they fail to capture displacement cascades and the initial evolution dynamics of radiation-induced defects under irradiation conditions. In this work, we focused on investigating both displacement cascade evolution and cascade interactions with crystal defects, such as grain boundaries and dislocations. Consequently, all-atom MD simulations were employed to investigate the defect production and the displacement cascade-induced atomic structural changes in PC FeCrAl alloys under neutron irradiation.

Clearly, a localized cascade within such a small region cannot change the overall temperature of the cladding. However, heat exchange occurs between these regions, and the cascade zone cools rapidly, almost maintaining the initial temperature of the system. This phenomenon effectively renders the surrounding atoms a physical thermostat for the cascade zone. At thermodynamic equilibrium, the average energy exchanged between the cascade region and its surroundings is balanced. Due to computational limitations, modeling a physical thermostat with over 10^25^ atoms is unfeasible using MD. To address this, periodic boundary conditions and the Nose–Hoover temperature-rescaling thermostat were implemented to simulate displacement cascades in PC FeCrAl alloys while reducing resource requirements.

All MD simulations were performed using the LAMMPS Molecular Dynamics Simulator [37]. The embedded atom method (EAM) potential for Fe-Cr-Al ternary systems developed by Liao et al. [38] was used to describe the interaction between atoms. It is worth noting that the potential parameters for FeCrAl alloys were determined by fitting to a set of experimental results and density functional theory (DFT) calculations. Previous studies have shown that this potential provides a realistic description of the properties of interstitials and vacancies [39,40], stacking fault energies and elastic constants [41], and was successfully applied to simulate irradiation damage in FeCrAl alloys [42,43]. Consequently, the displacement cascade simulations using this parameterized potential are expected to be capable for understanding the defect evolution and structural transformations in neutron-irradiated FeCrAl alloys.

Before the displacement cascades were performed, the energy minimization and the system relaxation process were performed to eliminate the residual thermal stresses in the alloy system for model initialization. Initial structures were relaxed using energy minimization via the conjugate gradient method. This was followed by relaxation in the canonical ensemble (NVT) for 500–2000 ps and subsequently in the isothermal–isobaric ensemble (NPT) at the target temperature and 0 Pa for another 500–2000 ps, with a time step of 0.001 ps. This preparation ensured structurally stable samples for subsequent primary radiation damage simulations. Displacement cascades were simulated under NPT conditions at temperatures ranging from 600 K to 1200 K and zero pressure, with a damping parameter 100 times the adaptive timestep. To limit atomic movement to 0.1 Å and atomic energy change to 2.5% of the PKA energy, the adaptive timestep was adjusted between 0.0001 and 2 fs. Each simulation ran for 50–200 ps, and adaptive timestep was adopted during the entire cascade process. Periodic boundary conditions were applied in all three spatial directions, and the Nose–Hoover thermostat was employed to realize the thermal equilibrium state of the crystalline systems.

Due to MD’s limitations in modeling inelastic collisions and the negligible energy loss from electron excitation, the common approximation assumes that the entire energy of the incident neutron is transferred to the PKA as kinetic energy. The simulation domain was divided into 8–36 regions, with one lattice atom randomly selected near the center of each region as the PKA. The entire simulation cell was repositioned under periodic boundary conditions to ensure that the PKA does not reach the cell boundaries. Then the PKA energy was given in the form of atomic velocity in a random direction, resulting in a wide irradiation dose range from 0.05 to 0.5 dpa. To maintain an equivalent dose when analyzing the effects of GS and temperature, the PKA energies ranged from 2.8 keV to 26.5 keV, as shown in Table 2. A displacement threshold of 0.245 nm, corresponding to the nearest-neighbor distance in FeCrAl alloys, was used to identify displaced atoms. Based on this, the radiation damage dose (*ϕ*) was quantified via atomic displacement analysis in OVITO [44]. PKAs were assigned initial velocities along the <135> crystallographic direction to avoid channeling effects common in low-index directions. Single-cascade simulations were conducted for doses below 0.2 dpa, while 2–4 statistically independent cascade events were performed at higher doses. Between successive cascade simulations, the system was equilibrated in the NPT ensemble at zero external pressure for 500–2000 ps to stabilize irradiation-induced defects and allow for recombination prior to subsequent irradiation events. Table 2 presents the parameters for the irradiation cascade simulations.

Our research group has been dedicated to investigating the high-temperature creep and high-temperature-irradiation creep behavior of FeCrAl alloys. Previous MD results on the high-temperature creep behavior of FeCrAl alloys can be found in references [39,40]. In order to study the high-temperature irradiation creep behavior of the alloy, the collision cascade processes of the alloy under different doses of irradiation damage were firstly simulated in this work. The evolution characteristics of grain boundary atoms and amorphous phases were analyzed, and the spatial distribution of point defects and dislocations before and after irradiation was compared to reveal the mechanisms of the generation and evolution of irradiation-induced defects. Furthermore, in subsequent studies, the high temperature creep behavior of the alloys with irradiation defects will be simulated to investigate the high temperature-irradiation creep performance and mechanisms of FeCrAl alloy under different doses. In this study, the displacement cascade simulation was performed in the NPT ensemble at zero pressure for two primary reasons: (i) Compared to temperature, pressure exerts a relatively minor and often negligible influence on the crystal lattice parameters. (ii) Setting zero pressure facilitates precise control over the pressure in the other two orthogonal directions during subsequent creep tensile simulations. This approach also helps avoid significant pressure fluctuations along the creep tensile direction.

### 2.3. Method for Determining the Evolution Mechanism of Defect Structures

As mentioned earlier, the interatomic potential developed by Liao et al. [38] was parameterized through fitting to experimental thermodynamic properties and DFT calculations, enabling accurate reproduction of crystal structures and defect behaviors in FeCrAl alloys. During initial irradiation, the PKA transfers kinetic energy to neighboring atoms, displacing them and initiating a cascade that generates FPs. The Wigner–Seitz cell method was employed to identify and count point defects in the crystalline structure, and the evolution of FPs throughout the cascade was analyzed. FPs evolve into vacancy and interstitial clusters, which drive mechanical degradation and can ultimately cause failure. Cluster analysis was conducted to evaluate defect reorganization during the annealing phase following irradiation of PC FeCrAl. Cluster size and number distributions were calculated using a cutoff distance-based method, where a cluster is defined as a group of particles connected directly or via intermediate particles. A cluster size of 1 indicates a single particle with no neighbors within the cutoff distance. Based on the results, a three-dimensional histogram of vacancy and interstitial cluster numbers as a function of irradiation dose and cluster size was constructed. To enhance visualization, three-dimensional spatial distribution maps of point defect clusters were generated, with coloring based on cluster type and size. In addition, the evolution and spatial distribution of GBs and amorphous structures were investigated using the CNA method and visualized with OVITO. Dislocation analysis (DXA) was performed to identify and classify different dislocation types based on Burgers vector analysis. Dislocation density was calculated using reconstructed atomic coordinates within the simulation volume.

Typically, CNA is designed to characterize the local structural environment, which provides an effective filtering method to classify atoms in crystalline systems. In this work, CNA was used to distinguish the structure of atoms, such as Other, BCC, FCC, HCP, ICO, and so on. The Boolean expression selection (“Structure Type = 0”) was used to select the type of “Other”, enabling quantitative analysis of the amorphous phase in the models. Representative CNA atomic snapshots were given to observe the amorphization transformation of neutron-irradiated FeCrAl alloys, shown in Section 3.5

## 3. Results and Discussion

### 3.1. Quantitative Analysis of Radiation Damage Dose

**Figure 2:**
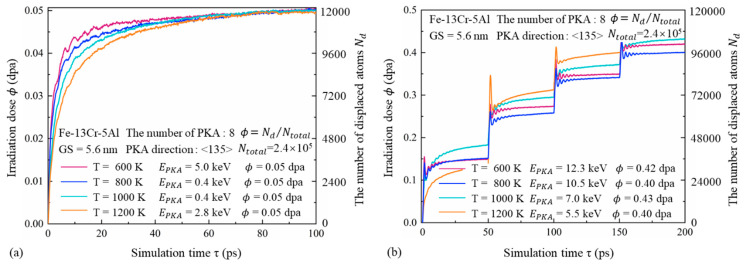
Time-dependent evolution of displaced atoms and radiation dose in Fe-13Cr-5Al samples with a GS of 5.6 nm under various temperatures and PKA energies for irradiation doses of (**a**) 0.05 dpa and (**b**) 0.40–0.43 dpa.

Figure 2 illustrates the number of displaced atoms Nd and the corresponding radiation dose *ϕ* as functions of time in the FeCrAl alloy with a GS of 5.6 nm, under different temperatures and PKA energies. A rapid increase in *ϕ* is observed during the ballistic and thermal-spike stages. However, after approximately 10 ps, corresponding to the onset of the quenching stage, the rate of increase slows significantly, eventually stabilizing during the annealing stage. As temperature increases, a lower PKA energy is required to achieve the same radiation dose, although it takes longer to reach the maximum dose. Elevated temperatures enhance the thermal motion of atoms, increasing their mobility and average displacement. This also reduces vacancy–interstitial recombination efficiency. Based on the single cascade results shown in Figure 2a, even when multiple PKAs are selected and higher initial energies are applied, the cumulative dose remains relatively low, not exceeding 0.20 dpa. To investigate the radiation damage behavior of FeCrAl at higher doses, 3–4 successive cascades were simulated, as illustrated in Figure 2b. In each cascade event, a sharp fluctuation in the number of displaced atoms is observed during the initial stage. As the cascade progresses into the quenching stage, the fluctuation amplitude gradually decreases, and the curve stabilizes after approximately 30 ps.

### 3.2. Displacement Cascade Evolution and Defect Number

**Figure 3:**
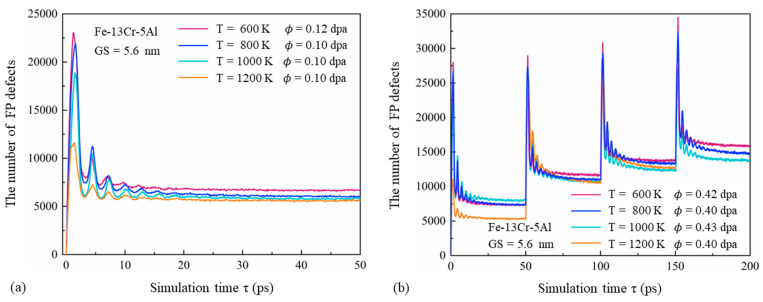
Time evolution of FP defects induced by cascades at various temperatures for irradiation doses of (**a**) 0.10–0.12 dpa and (**b**) 0.40–0.43 dpa.

**Figure 4:**
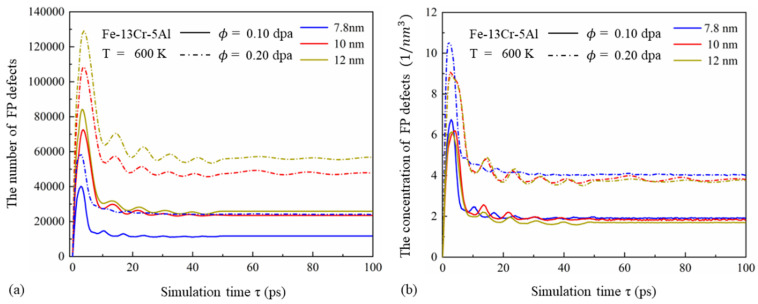
(**a**) Number and (**b**) concentration of FP defects versus time in Fe-13Cr-5Al cascades at 600 K with GSs ranging from 7.8 to 12 nm.

**Figure 5:**
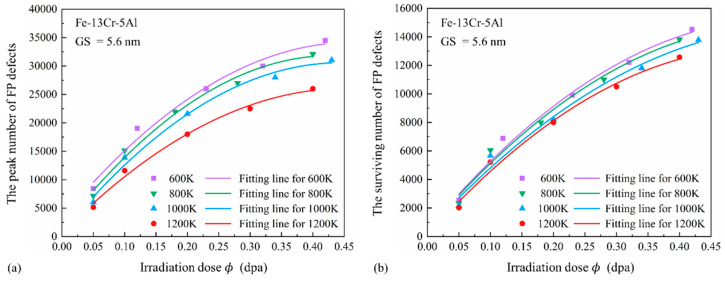
(**a**) Peak and (**b**) surviving FP defects versus irradiation dose in Fe-13Cr-5Al samples at temperatures ranging from 600 to 1200 K.

**Figure 6:**
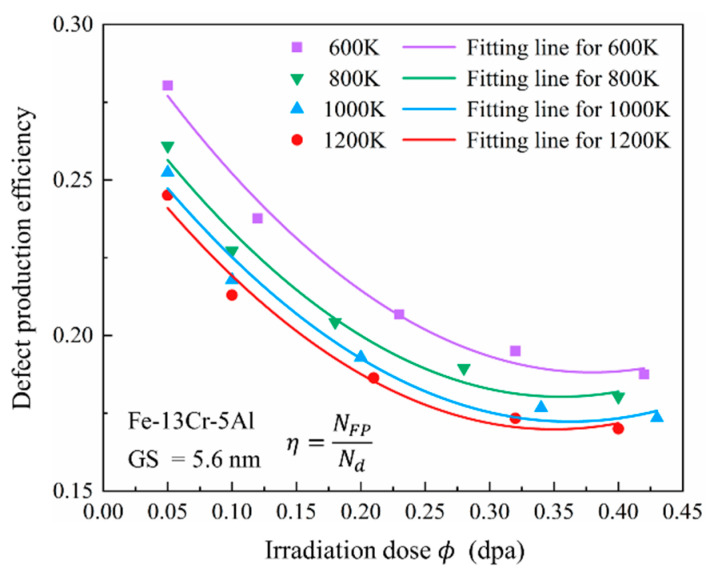
Defect production efficiency versus irradiation dose in Fe-13Cr-5Al alloys at 600–1200 K.

Displacement cascades typically proceed through four distinct stages: the ballistic stage, the thermal-spike stage, the quenching stage, and the annealing stage. In the ballistic stage, energetic neutrons interact with lattice atoms, initiating a series of collisions that persist until the energy of displaced atoms is no longer sufficient to sustain further collisions. These interactions generate a large number of displaced atoms. The energy of these atoms is then transmitted to surrounding atoms, forming a high-energy disordered core characteristic of the thermal-spike stage. As the cascade progresses, the energy of the displaced atoms gradually diminishes, enabling recombination with nearby vacancies. This marks the quenching stage, during which the number of FP defects significantly decreases compared to earlier stages. In the final stage—the annealing phase—lattice atoms diffuse and migrate, causing further recombination and stabilization of the FP defect size and distribution. Depending on the irradiation conditions, the annealing stage can persist from nanoseconds to months, as mobile defects continue to evolve. In this study, the ballistic, thermal-spike, quenching, and early annealing stages in PC FeCrAl were simulated under various conditions. The time evolution of FP defects is discussed based on Figure 3 and Figure 4 in the following. Additionally, the effects of irradiation dose and temperature on the peak number of FP defects (*N_peak_*), the number of surviving FP defects (*N_FP_*), and defect production efficiency (*η*) are analyzed, as shown in Figure 5 and Figure 6.

#### 3.2.1. Cascade Evolution Under Different Irradiation Doses

##### Temperature Effect

Figure 3 presents the temporal variation in FP defects in displacement cascades of PC Fe-13Cr-5Al with a GS of 5.6 nm under varying temperature and dose conditions. For each temperature condition, despite differences in absolute values, the general trends are consistent. Within the first 0–1 ps, the number of FP defects increases sharply and reaches a maximum. Unlike the behavior observed in SC FeCrAl [12], the number of FP defects subsequently decreases and exhibits fluctuations during the quenching stage. These fluctuations can be attributed to the interconversion between the kinetic and potential energy of displaced atoms. When kinetic energy peaks, the number of FPs reaches a minimum, and vice versa. As the cascade continues, the energy of displaced atoms decreases, leading to reduced fluctuations and a more stable FP count. At higher temperatures, the peak occurs earlier, and both the quenching and annealing stages are shortened. As a result, the peak and surviving numbers of FPs are lower. This behavior can be explained by the reduced initial PKA velocity required to maintain a constant dose at elevated temperatures, as well as the enhanced thermal vibration of atoms that facilitates recombination with nearby vacancies, thereby reducing defect production efficiency.

##### Grain Size Effect

Figure 4a,b show the time evolution of the number and concentration of FP defects under different GSs. At a constant dose, the total number of FP defects increases significantly with GS. This is due to the fact that larger GSs contain more PKAs with higher initial energy (see Table 1), resulting in larger cascade volumes. However, the concentration of FPs decreases as GS increases, with this trend becoming more pronounced at higher doses. This can be attributed to the reduced volume fraction of GBs, which limits the absorption of displaced atoms and enhances recombination and annihilation during the quenching and annealing stages. Similar results have been reported by Zhang et al. [45] with a neutron-irradiated BCC Fe-Cr alloy using MD simulations. Additionally, higher PKA energy associated with larger grains leads to more compact cascade regions, increasing the likelihood of recombination. As observed in Figure 3 and Figure 4, higher irradiation doses result in delayed peak FP numbers, greater fluctuation amplitudes, and longer quenching stages. These effects are caused by the more intense atomic collisions triggered by high-energy PKAs, which increase the FP peak and prolong recombination time.

#### 3.2.2. Influence of Irradiation Dose and Temperature on Defect Number

##### Peak and Surviving FP Defects

Figure 5a shows the relationship between the peak number of FP defects (*N_peak_*) and irradiation dose (0.05–0.5 dpa) at various temperatures for Fe-13Cr-5Al samples with a 5.6 nm GS. A positive correlation is observed between *N_peak_* and dose, although the slope of the fitted curve gradually decreases. For low irradiation doses, single-cascade simulations are used, while, at doses above 0.2 dpa, two to four cascades are conducted to improve computational efficiency and accuracy. Under identical GS conditions, all cascades are performed using the same PKA direction and count. The observed increase in *N_peak_* can be attributed to both higher PKA energies and a greater number of cascades. At the same dose, elevated temperature reduces *N_peak_*, likely because lower PKA energy is required, resulting in smaller cascade zones. Figure 5b shows that the number of surviving FP defects (*N_FP_*) increases approximately linearly with dose. For each temperature, a 1 dpa increase corresponds to an approximate rise of 31,430 FPs. This increase is primarily driven by the enhanced PKA energy and the multiple cascades at higher doses. As temperature rises, the values of *N_FP_* decrease slightly, possibly due to the heightened motion of atoms, which strengthens the recombination effect and lowers the number of stable FPs.

##### Defect Production Efficiency

Defect production efficiency (*η*) represents the number of surviving FPs after each atom in the crystalline system is dislocated once and is a key parameter in evaluating radiation damage. Values of *η* during the annealing stage provide insight into the severity and extent of radiation effects. As shown in Figure 6, *η* decreases first with each increasing dose and tends to be stable with a turning point of 0.30 dpa. This trend is likely due to increasing PKA energy and the number of cascades at higher doses, which introduce more sub-cascades and extend both quenching and annealing stages, thus promoting recombination. Furthermore, each cascade leaves behind residual defects, which may recombine with defects generated in subsequent cascades, thereby lowering *η*. Elevated temperatures also reduce *η*, as enhanced atomic mobility promotes recombination between vacancies and interstitials.

### 3.3. Distribution of Point Defects and Defect Clusters

**Figure 7:**
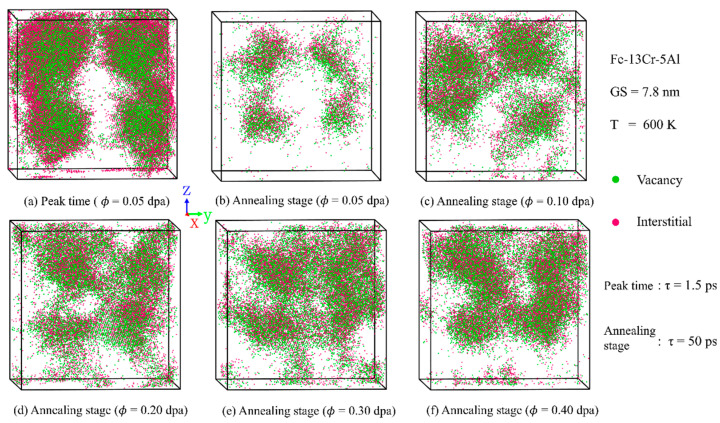
Distribution snapshots of point defects in PC Fe-13Cr-5Al at (**a**) peak and (**b**–**f**) annealing stages under varying irradiation doses.

**Figure 8:**
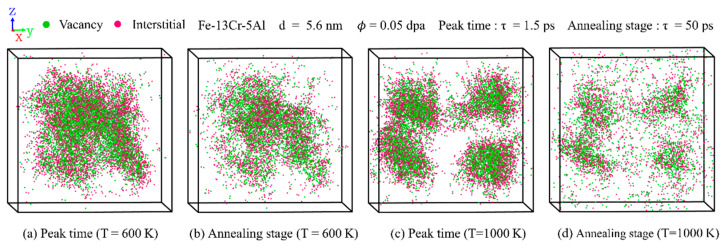
Distribution snapshots of point defects in PC Fe-13Cr-5Al at peak and annealing stages under different temperatures: (**a**,**b**) 600 K and (**c**,**d**) 1000 K at a dose of 0.05 dpa.

**Figure 9:**
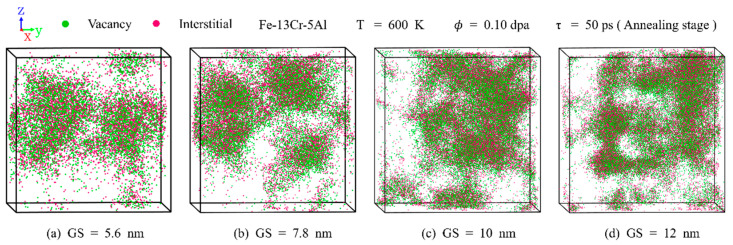
Distribution snapshots of point defects in PC Fe-13Cr-5Al under GSs of (**a**) 5.6 nm, (**b**) 7.8 nm, (**c**) 10 nm, and (**d**) 12 nm at the annealing stage with a dose of 0.10 dpa.

**Figure 10:**
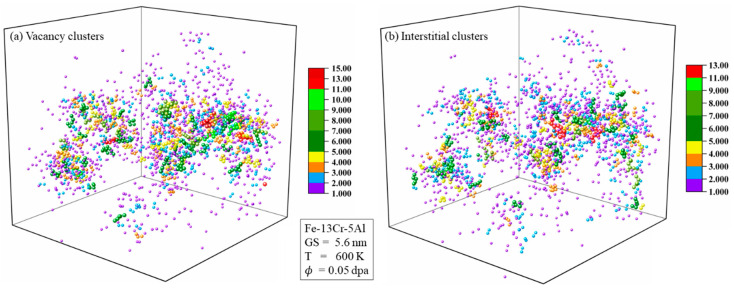
Distribution snapshots of (**a**) vacancy clusters and (**b**) interstitial clusters in PC Fe-13Cr-5Al at the annealing stage with a dose of 0.05 dpa.

**Figure 11:**
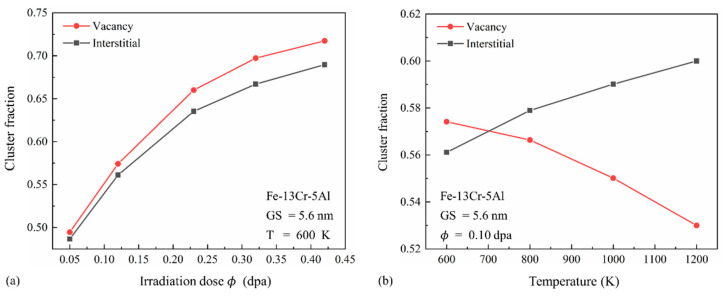
Clustering fraction of vacancies and interstitials for (**a**) varying doses from 0.05 to 0.45 dpa at 600 K and (**b**) different temperatures (600–1200 K) at a dose of 0.10 dpa in PC Fe-13Cr-5Al with a GS of 5.6 nm.

**Figure 12:**
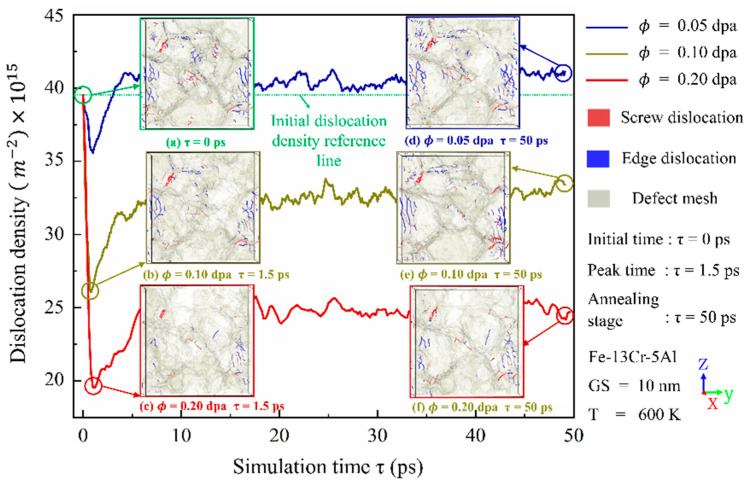
Time evolution of dislocation density under different irradiation doses, along with representative distribution snapshots of dislocation defects.

Point defects, specifically interstitials and vacancies, are the primary defects generated during the initial stages of irradiation. These defects aggregate and evolve into defect clusters, contributing to significant changes in the mechanical properties of crystalline materials. Therefore, the characteristics of point defects and defect clusters are crucial in evaluating the damage evolution of irradiated PC FeCrAl alloys. The spatial distribution and temporal evolution of these irradiation-induced point defects are analyzed based on Figure 7, Figure 8 and Figure 9. The defect clustering fraction is defined as the ratio of the number of defects forming a cluster to the total number of defects of the same type. The distribution and clustering fraction of vacancy and interstitial clusters at the end of the cascade process are presented in Figure 10, Figure 11 and Figure 12.

#### 3.3.1. Point Defects

Figure 7 presents the distribution of vacancies and interstitials in PC Fe-13Cr-5Al at the peak and annealing stages under various irradiation doses. Vacancies are primarily located near the cascade core and surrounded by interstitials, likely due to the higher migration energy of vacancies compared to interstitials. As FP recombination progresses, the cascade volume observed during the annealing stage becomes significantly smaller than that at the peak. Additionally, higher irradiation doses result in more intense atomic collisions and increased average atomic displacements, thereby enlarging the cascade volume. Similar observations can also be found in the study of other BCC metals such as tungsten [46], using atomistic simulations. This behavior is attributed to the greater initial energy of PKAs and an increased number of sub-cascades under high-dose conditions. High-energy PKAs can move farther away and transfer energy to surrounding atoms, initiating more sub-cascades, and more atoms are dislocated by the collision. Moreover, due to the channeling effect of high-energy PKAs, cascade regions become interconnected when the dose exceeds 0.10 dpa.

Figure 8 compares the distribution of point defects under varying temperature conditions. With the rise in temperature, cascade regions evolve from interconnected to isolated structures. The number of surviving defects decreases with temperature due to enhanced recombination effects. This is also consistent with previous ion irradiation experiments [47] on commercial FeCrAl alloys, which indicate that, with increasing temperature, the point defect density is reduced due to annealing-induced recovery effects on atomic displacement. Figure 9 illustrates the influence of GS on the distribution of point defects. The total number of surviving defects increases notably with GS. This trend is attributable to the higher energy and quantity of PKAs in larger grains under the same dose. With increasing GS, cascade regions transition from regular spherical shapes to irregular shapes. This behavior can be explained by the larger average atomic displacement and stronger channeling effect associated with higher-energy PKAs.

#### 3.3.2. Defect Clusters

Figure 10 presents the spatial distribution of vacancy and interstitial clusters after the cascade reaches stability, with defects color-coded by cluster size. Clusters that have a size greater than 3.0 are distributed near the cascade core, while clusters with sizes of 1.0 or 2.0 are scattered in the peripheral region. This distribution arises because atomic collisions are more intense in the core region, where the defects are closer to each other and more likely to form larger clusters. And, the larger the cluster size, the higher the formation energy, and once formed, it is less likely to migrate. The number and size of clusters are negatively correlated, with larger cluster sizes resulting in fewer clusters. This can also be attributed to the higher formation energy of the clusters with larger sizes.

The effects of temperature and irradiation dose on the clustering fractions of vacancies and interstitials are shown in Figure 11. As illustrated in Figure 11a, vacancy clustering fractions exceed those of interstitials, particularly at high doses. Previous studies indicate that, while vacancies have lower formation energy, their migration energy is higher than that of interstitials. Consequently, vacancy clusters form more readily but exhibit limited mobility, whereas interstitial clusters are generally more mobile but less likely to form. During the collision phase, vacancies—being densely concentrated in the cascade core—are more likely to form multi-vacancy clusters. In contrast, interstitials are more spatially dispersed and tend to cluster through diffusion-driven aggregation. Under the high-dose condition, increased PKA initial energy intensifies atomic collisions, resulting in a higher density of vacancies in the core, thus forming more vacancy clusters. However, the increase in PKA energy makes the average distance between the interstitials longer, and they are less likely to meet with each other and form clusters. Figure 11 also shows that the increase in dose enhances the clustering fraction of both defect types, primarily due to increased defect numbers and cascade overlap. In Figure 11b, the clustering fraction of vacancies decreases with temperature, whereas that of interstitials increases. This is because, at lower temperatures, vacancies remain concentrated near the cascade core, while interstitials are dispersed. Although the migration energy of interstitials is lower than vacancies, clustered vacancies outnumber clustered interstitials at low temperatures because the defects can hardly reach their migration energy. Elevated temperatures enhance atomic motion, facilitating interstitial interactions and cluster formation. Simultaneously, high temperatures destabilize vacancy clusters, leading to their decomposition. As a result, the interstitial clustering fraction surpasses that of vacancies at elevated temperatures.

### 3.4. Evolution of Dislocation Defects

**Figure 13:**
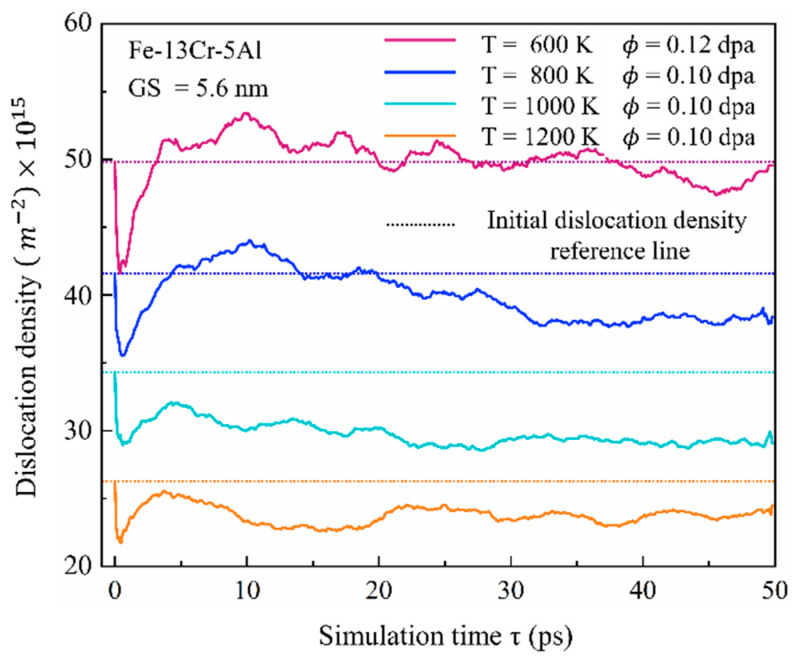
Time evolution of dislocation density at various temperatures under an irradiation dose of 0.10–0.12 dpa.

Irradiation-induced point defects interact with existing dislocations in crystalline materials, leading to changes in both their density and spatial distribution and thus affecting the mechanical properties. Figure 12 and Figure 13 depict the time evolution of dislocation density under varying temperatures and irradiation doses, along with representative snapshots of dislocation structures at the peak and annealing stages. It can be seen that, despite variations in absolute values, the general trend of each curve is consistent: dislocation density initially decreases, then increases, and eventually stabilizes. Compared with unirradiated models, a slight reduction in the number of dislocations is observed at the peak stage. This reduction may be attributed to the enhanced mobility of dislocations at elevated temperatures, which promotes interactions with GBs, leading to their absorption and subsequent annihilation. After the cascade process enters the annealing stage, the temperature gradually decreases, and dislocation density begins to increase. Figure 12 also shows that higher irradiation doses result in a pronounced reduction in dislocation density, in agreement with experimental observations [48] of austenitic stainless steel under ion irradiation. This phenomenon is likely due to intensified atomic collision processes and enhanced dislocation movement, which facilitate dislocation absorption at GBs.

Figure 13 reveals that the number of dislocations surviving in the annealing stage decreases progressively with increasing temperature. This trend can be attributed to the thermal activation of dislocation motion, which enhances their likelihood of annihilation. The same phenomenon has also been found in earlier experimental results of Massey et al. [49] on neutron-irradiated commercial FeCrAl alloy C35M. Therefore, both temperature and dose play pivotal roles in determining the dislocation density at peak time and the end of cascades, thereby affecting the mechanical properties of PC FeCrAl alloys. The relatively minor fluctuations in the curves of Figure 12 and Figure 13 during the annealing stage indicate that the dislocation distribution in this phase plays a critical role in the overall microstructural evolution of FeCrAl alloys, including atomic diffusion and GB sliding processes.

**Figure 14:**
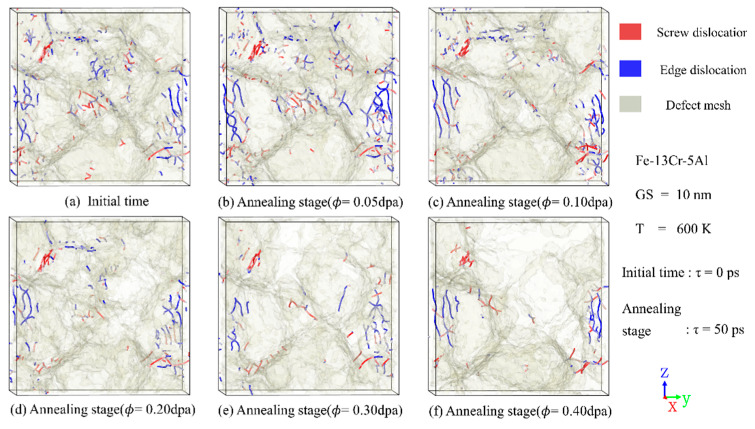
Snapshots showing the distribution of dislocations and defect meshes in PC Fe-13Cr-5Al alloys at (**a**) initial time and (**b**–**f**) annealing stage under different dose conditions.

**Figure 15:**
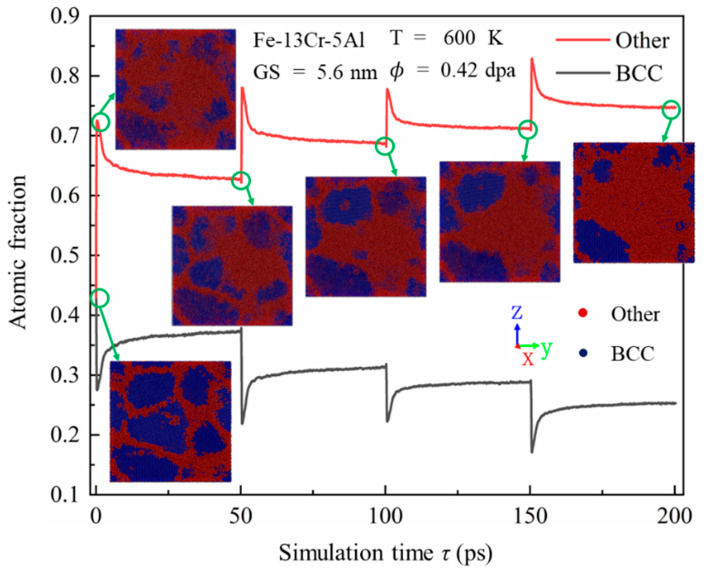
Time evolution of atomic fractions for BCC and amorphous structures in PC Fe-13Cr-5Al alloys, along with atomic snapshots colored by crystal structure through CNA at different simulation times.

**Figure 16:**
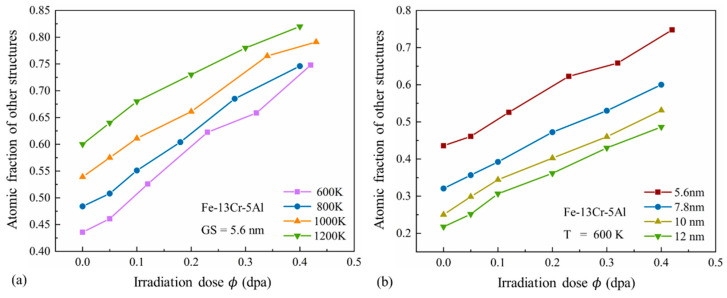
Atomic fraction of amorphous structures versus irradiation dose under (**a**) different temperatures and (**b**) different GS conditions.

**Figure 17:**
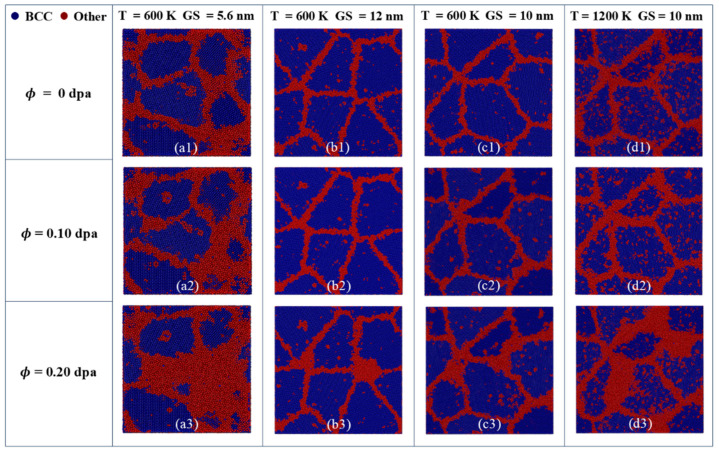
CNA-colored atomic snapshots showing structural evolution in PC Fe-13Cr-5Al under different temperature and GS conditions for irradiation doses of (**a1**-**d1**) 0 dpa, (**a2**-**d2**) 0.10 dpa and (**a3**-**d3**) 0.20 dpa.

Figure 14 presents the spatial distribution of dislocations at the annealing stage under different dose conditions. Irradiation has an impact on the density and distribution of dislocations, but the trend and magnitude are related to the radiation dose. At low doses, a slight increase in dislocation density was noted after irradiation compared to those before. However, at doses exceeding 0.2 dpa, the amount of dislocations decreases significantly, possibly because the dislocation is swallowed by the amorphous atoms, and the amorphous phase is formed, as can be seen in Figure 15, Figure 16 and Figure 17.

### 3.5. Formation and Evolution of Amorphous Structures

As discussed previously, irradiation can lead to significant microstructural changes in PC FeCrAl alloys. In addition to introducing numerous point defects and defect clusters, leading to changes in dislocation distribution and density, irradiation may also induce a transformation from crystalline to amorphous structures. This transformation is an important finding regarding the irradiation damage mechanisms of PC FeCrAl alloys. Figure 15 shows the time evolution of the atomic fractions of BCC and amorphous structures from the onset of displacement cascades to 200 ps. To achieve high irradiation doses over a short timescale, four consecutive cascade simulations were performed. As the cascade proceeds, a similar change trend over time is observed between the fraction of amorphous structures and the number of FPs, indicating that irradiation-induced point defects significantly influence amorphous phase formation. With time, there is a gradual rise and fall in the atomic proportion of a BCC and amorphous structure in the model, reflecting the dynamic nature of structural transformations during the cascade process. After four consecutive irradiations, the atomic fractions stabilize at approximately 25% BCC and 75% amorphous structures. To more intuitively show the evolution of the amorphous structure, Figure 15 also provides representative snapshots of atomic arrangement colored by crystal structure through a CNA. After the first cascade approaches a relatively stable state at approximately τ = 50 ps, amorphous regions adjacent to the GBs appear in the crystal. Similar behaviors were observed by Wan et al. [50] in ion-irradiated nano-amorphous Ti-Si-N composite film with grain sizes from 5 nm to 12 nm. This is likely due to the preferential accumulation of point defects on both sides of the GB, which leads to significant GB thickening. As the accumulated dose rises to a constant value, the crystalline regions around GBs progressively transform into an amorphous state. These observations are consistent with the recent experimental results [51] for Pt thin films with 18 nm thickness, which indicate that irradiation-induced defects resulted primarily in localized roughening of the GB thickness and changes in the local atomic structure of the GB.

The effects of temperature and GS on the amorphous process of FeCrAl alloys are further explored. Figure 16 shows the relationship between the atomic fraction of amorphous structures and the irradiation dose under different temperatures and GS conditions. It can be observed that the atomic fraction of amorphous structures increases progressively with dose and becomes significantly higher at elevated temperatures or smaller GS. This suggests that higher doses and temperatures accelerate the amorphization process, while smaller GSs promote more extensive amorphization of the grains in PC FeCrAl alloys.

Figure 17 provides CNA-colored atomic arrangement snapshots under different irradiation doses, temperatures, and GS conditions. At low doses and temperatures, the grain size and shape remain relatively unchanged, despite a slight increase in the thickness of the GB layer. Under higher temperatures and dose conditions, a number of atoms near the GB transit from BCC to the amorphous phase, resulting in noticeable changes in grain shape and size. From Figure 17(b3,c3,d3), at a dose of 0.2 dpa and a temperature of 600 K, only a small amount of amorphous phase is formed and gathered at GBs. In contrast, as the temperature rises to 1200 K, a pronounced amorphous transformation occurs in the crystal. Figure 17(a1–a3,b1–b3) further reveal that larger GSs require higher doses to develop extensive and continuous amorphous regions. It suggests that increasing the GS can greatly enhance the irradiation resistance of PC FeCrAl alloys, which is similar to the research of nano-amorphous TiSiN composite coatings [50]. In summary, irradiation will lead to amorphous phase formation inside the grains, with the area of amorphous regions expanding as dose increases. The extent of amorphization is further amplified by rising temperature or decreasing GS.

## 4. Conclusions

In this study, the displacement cascade process in PC FeCrAl alloys was simulated under different doses of irradiation and different levels of temperature and GS. The formation and evolution of point defects, dislocations, GBs, and amorphous structures at different cascade stages were thoroughly analyzed. In addition, the microstructural damage mechanisms governing the irradiation response of the alloy were comprehensively discussed. Based on the presented analysis, the following statements can be drawn:

(1) Temperature, irradiation dose, and GS have an important impact on the number and morphology of peak and surviving FPs. Temperature decreases the number of FPs, and this trend becomes more pronounced at higher doses. While the number of FPs increases substantially with GS, the FP concentration decreases slightly. With increasing dose and GS, or decreasing temperature, cascade regions transit from being independent to interconnected, and their shapes evolve from spherical to polymorphic.
(2) FPs generated by cascades tend to aggregate, forming clusters. The cluster fraction goes up for both interstitials and vacancies with the increase in dose, and the cluster fraction of interstitials increases with temperature, while the vacancy fraction decreases. At lower temperatures, clustered vacancies are more than the clustered interstitials because the vacancies are close to each other, and interstitials are far from each other. However, elevated temperatures enhance interstitial mobility, enabling them to cluster more effectively and eventually surpass vacancy clusters.
(3) FPs interact with existing dislocations, altering dislocation density and distribution during the early stages of irradiation damage in PC FeCrAl. Dislocation density decreases significantly with increasing dose and temperature. At lower doses, a slight increase in dislocation density is observed compared to the unirradiated model, but at higher doses, the dislocation density decreases markedly.
(4) FPs preferentially migrate to and are absorbed at GBs, leading to a noticeable increase in GB thickness with rising dose. When the accumulated dose exceeds a critical threshold, regions near GBs undergo a transformation into an amorphous phase. The amorphization process accelerates with higher dose and temperature, and smaller GS results in a more extensive amorphous transformation.

In PC FeCrAl alloys, a great number of FPs and defect clusters are introduced by irradiation, and the interactions of FPs and dislocations result in variations in dislocation density. Furthermore, irradiation can induce the transformation of crystalline regions into amorphous structures—an important finding for understanding the fundamental mechanisms of irradiation damage in FeCrAl alloys.

## Figures and Tables

**Table 1:** Parameters of the polycrystalline (PC) Fe-13Cr-5Al models with different grain sizes.

Grain Size (nm)	The Number of Atoms (×10^5^)	Fraction of GB * Atoms (%)	Simulation Box Size (nm)	The Number of Grains
5.6	2.4	24.2	14.1	16
7.8	4.8	17.4	17.9	12
10.0	10.1	13.7	22.9	12
12.0	12.2	12.0	24.4	8

* Grain boundary (GB).

**Table 2:** Parameters of the model and irradiation cascade simulations.

Grain Size (nm)	5.6	7.8	10	12
Relaxation time of NVT/NPT ensembles (ps)	500	1000	1500	2000
The number of PKA per cascade	8	16	27	36
The initial energy of PKA (keV)	2.8–12.3	4.8–24.6	5.9–29.0	5.4–26.5
Temperature (K)	600, 800, 1000, 1200			

## Data Availability

Data are contained within the article.
